# Delayed Diagnosis of Aicardi-Goutières Syndrome in a 10-Year-Old Female Child With TREX1 Mutation: A Case Report

**DOI:** 10.7759/cureus.79730

**Published:** 2025-02-26

**Authors:** Leen Al Zayer, Mustafa Al Zayer, Abdulrahman Alrujaib, Asal Buhasan, Raafat Hamad Seroor H Jadah

**Affiliations:** 1 School of Medicine, Royal College of Surgeons in Ireland, Busaiteen, BHR; 2 Medicine, St Mary's Hospital, Newport, GBR; 3 Pediatric Neurology, Bahrain Defence Force Hospital, Riffa, BHR

**Keywords:** aicardi goutières syndrome, genetic disease, neurology, pediatrics, trex1 mutation

## Abstract

Aicardi-Goutières syndrome (AGS) is a rare hereditary autoinflammatory disease of subacute encephalopathy, characterized by a wide range of neurological and extra-neurological manifestations, primarily affecting the brain and skin. Key features include increased expression of interferon-stimulated genes (ISGs), acquired microcephaly, dystonia, spasticity, chilblains, and panniculitis. Radiological findings include cerebral calcifications, leukodystrophy, cerebral atrophy, and cerebrospinal fluid abnormalities such as chronic lymphocytosis and elevated interferon-alpha (INF-α) levels. Seven pathogenic genes have been identified in association with AGS. The management of AGS is primarily supportive, as there is currently no definitive cure for the condition. The primary goals are to address symptoms, improve quality of life, and prevent complications. The aim of this study was to emphasize the importance of early diagnosis of this rare genetic condition, as timely identification enables prompt intervention and management. Early diagnosis improves clinical outcomes, enhances the quality of life for affected individuals, and provides valuable guidance for family planning and genetic counseling.

## Introduction

Aicardi-Goutières syndrome (AGS) is a rare hereditary autoinflammatory disease of subacute encephalopathy, characterized by a wide range of neurological and extra-neurological manifestations, primarily affecting the brain and skin [1]. Typical clinical manifestations include acquired microcephaly, dystonia, spasticity, chilblains, panniculitis, with radiological findings of cerebral calcifications, leukodystrophy, cerebral atrophy and, cerebrospinal fluid abnormalities, including chronic lymphocytosis and elevated interferon (INF)-α levels [1].

Patients with AGS exhibit increased expression of interferon-stimulated genes (ISGs) in their peripheral blood. Monitoring ISG expression can serve as a valuable biomarker for assessing disease activity [1]. To date, seven pathogenic genes have been identified as being linked to AGS: *TREX1* (AGS1), *RNASEH2B* (AGS2), *RNASEH2C* (AGS3), *RNASEH2A* (AGS4), *SAMHD1* (AGS5), *ADAR1* (AGS6), and *IFIH1* (AGS7) [1].

AGS classically presents in infancy as severe neurological dysfunction, with progressive microcephaly, spasticity, psychomotor delay, and potential early childhood mortality [2]. Moreover, its association with excessive type I INF production contributes to systemic manifestations, including thrombocytopenia, hepatosplenomegaly, and abnormal liver function tests. These findings, along with similar radiological features, are also observed in various congenital infections, which blurs the distinction between these two conditions [2]. Consequently, AGS should be considered in neonates presenting with symptoms indicative of a congenital infection accompanied by sterile pyrexia. Furthermore, there is a notable overlap in clinical features between AGS and systemic lupus erythematosus (SLE), a more common condition that is also linked to abnormal upregulation of type I INF [2].

Therefore, early recognition and accurate diagnosis of AGS remains a significant challenge for clinicians. Further research is needed to develop effective diagnostic tools and treatment strategies for these patients.

## Case presentation

A 10-year-old girl, the first child of consanguineous parents who are first-generation cousins, was born after a full-term pregnancy complicated by hypoxic-ischemic encephalopathy (HIE) following a 20-minute arrest. She developed spastic quadriplegic cerebral palsy (CP) with hyperreflexia and global developmental delay (GDD). The patient had multiple intractable seizures, including daily focal unaware and generalized tonic-clonic seizures. Recent improvements in seizure control have been observed with current antiepileptic therapy, which includes clobazam 10 mg orally once daily for the past year, phenobarbitone 15 mg orally twice daily for the past three years, lamotrigine 25 mg orally twice daily for the past two years, and lacosamide 40 mg orally twice daily for the past two years. She is currently under regular follow-up with a pediatric neurologist and is showing improvement with well-controlled seizures and no further seizure activity. Family history includes consanguinity and a similar condition in the patient's younger brother, who was genetically confirmed to have the same gene mutation. Past admissions include recurrent aspiration pneumonia, necessitating chest physiotherapy, and recurrent breakthrough seizures. 

**Figure 1:**
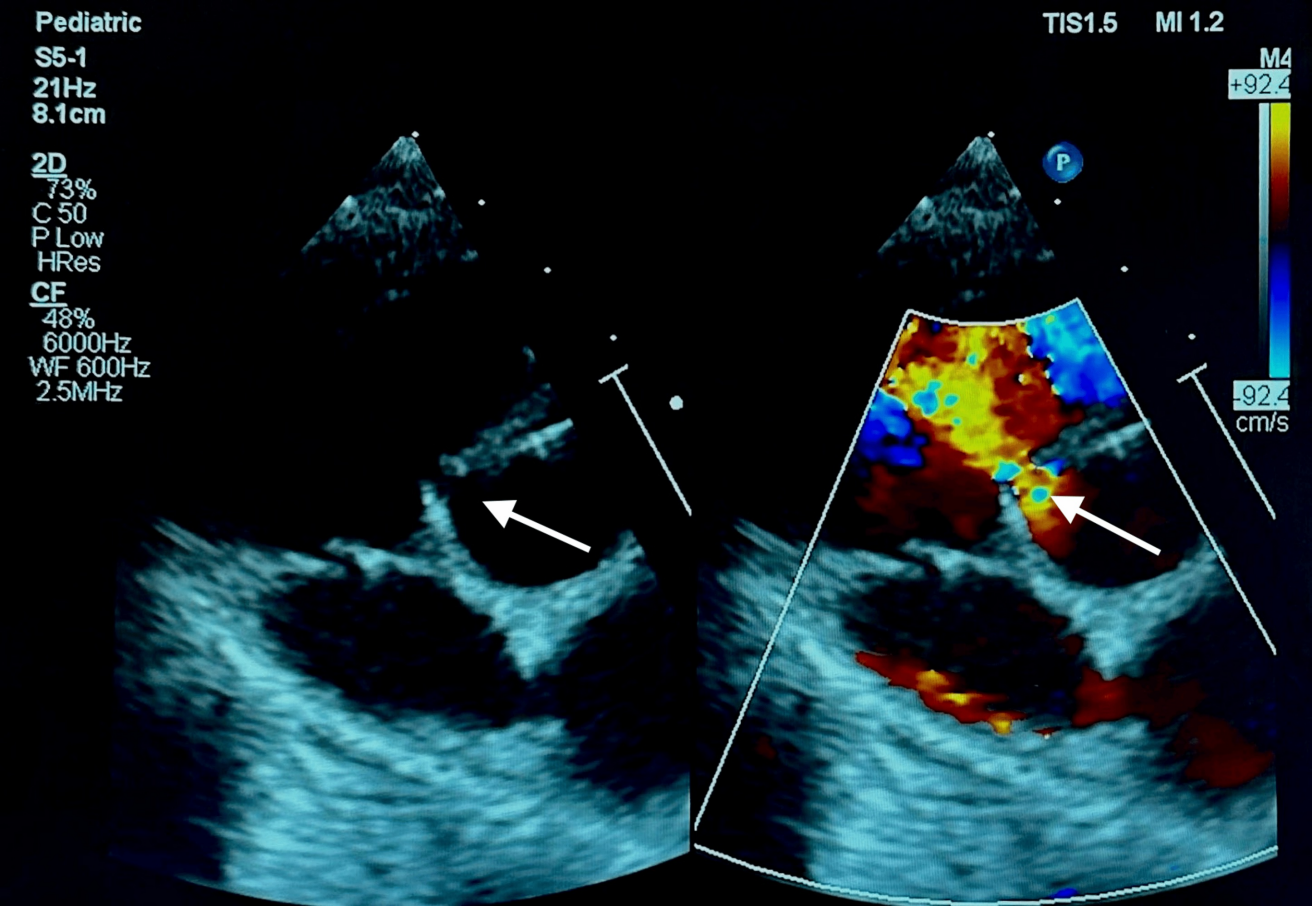
Echocardiogram revealing small, 2mm, perimembranous ventricular septal defect with mild mitral and tricuspid regurgitation and an intact atrial septum. Left ventricular systolic function was preserved with no evidence of hypertrophic obstructive cardiomyopathy.

Physical examination demonstrates gross delay, generalized spasticity, and reduced power with brisk deep tendon reflex (DTR) of +4 and ankle clonus. There are no dysmorphic features, neurocutaneous skin lesions, or cerebellar signs. The remainder of the examination demonstrated a grade III ejection systolic murmur. Echocardiography, as seen in Figure 1, revealed a ventricular septal defect, mild mitral and tricuspid regurgitation, with preserved left ventricular systolic function.

She is currently being managed for her congenital heart disease (CHD) with atenolol 25 mg orally once daily and furosemide 20 mg orally once daily, as recommended by the pediatric cardiologist, for the past six months. 

Following whole exome sequencing (WES), she was diagnosed with AGS; Table 1 was extracted from the WES report showing the identified gene. While dominant inheritance is suspected due to the patient's younger brother being diagnosed with the disease, we were unable to definitively confirm the mode of inheritance or rule out a de novo mutation as both parents refused genetic testing. 

**Table 1:** Whole exome sequencing identifying a heterozygous pathogenic variant in the TREX1 gene

Gene (Transcript)	Variant	Genomic Position	Zygosity	Disorder (OMIM)	Classification
*TREX1* (NM_033629.6)	c.58dup (p.Glu20GlyfsTer82)	GRCh37 (chr3-48508110 T>TG)	Heterozygous	Aicardi-Goutieres syndrome 1, dominant and recessive (OMIM#225750)	Pathogenic

## Discussion

AGS is a rare genetic disorder, with an estimated prevalence of one in 10,000 live births, although the exact prevalence remains unknown. The condition shows no gender preference [3]. To date, seven pathogenic genes have been identified as being linked to AGS. Our patient was diagnosed at the age of 10 with a mutation in the *TREX1* gene. 

According to recent literature, *TREX1 *and *RNASEH2B* are the most implicated genes among these seven variants, accounting for 17% and 35% of reported cases, respectively [4]. AGS can be classified based on the severity of its features and the age of symptom onset, typically categorized into early-onset and late-onset forms. Variants in the *TREX1*, *RNASEH2A*, and *RNASEH2C* genes are associated with the early-onset or "classic" form of AGS, characterized by more severe symptoms and early neurological decline [4,5]. Conversely, mutations in the *RNASEH2B*, *SAMHD1*, *IFIH1*, and *ADAR1* genes are more commonly linked to the later-onset form, which tends to present with milder symptoms and slower disease progression [4,5].

A study by Adang et al. investigating the developmental outcomes of AGS based on genotype revealed significant differences in severity among genetic variants [4]. Children with *TREX1-*related AGS were found to be the most severely affected, while those with mutations in *SAMHD1*, *ADAR1*, and *IFIH1* demonstrated the most advanced developmental achievements overall. The *TREX1*-related form of AGS was statistically distinct from other genotypes in terms of earlier presentation, with an average onset age ranging from one to three months, as well as a delayed acquisition of key milestones, such as head control, sitting, rolling over, smiling, and saying nonspecific words like "mama" or "dada” [4].

Identifying the molecular cause is therefore essential for more personalized management and risk assessment in family members. Comprehensive genetic testing methods, such as WES, are increasingly being used alongside traditional diagnostic approaches, including physical examinations, imaging studies, and biochemical tests, to confirm genetic mutations [6].

The management of AGS is primarily supportive, as there is currently no definitive cure for the condition. The primary goals are to address symptoms, improve quality of life, and prevent complications. Management strategies are tailored to the specific manifestations of the disease, which can vary widely among affected individuals [7]. Epilepsy is a common feature, affecting approximately one-third of patients, with tonic seizures accounting for 69% of seizure types. Consequently, management includes the use of anticonvulsant therapy to control seizures. In addition to managing seizures, other supportive measures, such as respiratory assistance and nutritional support, are often required. Additionally, AGS patients may present with thrombocytopenia, which may require platelet transfusion [7]. Theoretically, anti-IFN monoclonal antibodies or inhibitors could be used to treat AGS [7]. Moreover, JAK inhibitors such as baricitinib are under investigation, where a recent study demonstrated a reduction in IFN levels and improved cognitive function and communication skills, while gross motor deficits persisted [8].

## Conclusions

This case highlights the diagnostic challenges of AGS, particularly in patients presenting with complex neurological manifestations. It underscores the importance of considering AGS in patients with unexplained neurological deterioration, especially in the context of consanguinity and a positive family history. Early diagnosis through comprehensive genetic testing, such as WES, is crucial for appropriate management, which focuses on supportive care, including seizure control, respiratory support, and addressing other complications. While there is no cure for AGS, future research is needed to explore the therapeutic potential of emerging treatments such as JAK inhibitors, which may alter the course of the disease.
